# STAT3 and SOX-5 induce BRG1-mediated chromatin remodeling of RORCE2 in Th17 cells

**DOI:** 10.1038/s42003-023-05735-9

**Published:** 2024-01-03

**Authors:** Xian Wang, Chao Han, Di Yang, Jian Zhou, Hui Dong, Zhiyuan Wei, Shuai Xu, Chen Xu, Yiwei Zhang, Yi Sun, Bing Ni, Sheng Guo, Jingbo Zhang, Tingting Zhao, Xiangmei Chen, Jie Luo, Yuzhang Wu, Yi Tian

**Affiliations:** 1 Institute of Immunology, Third Military Medical University (Army Medical University), 400038 Chongqing, People’s Republic of China; 2 Department of Immunology, Medical College of Qingdao University, 266071 Qingdao, Shandong People’s Republic of China; 3 The First Affiliated Hospital, Third Military Medical University (Army Medical University), 400038 Chongqing, People’s Republic of China; 4 The Second Affiliated Hospital, Third Military Medical University (Army Medical University), 400037 Chongqing, People’s Republic of China; 5 Department of Pathophysiology, Third Military Medical University (Army Medical University), 400038 Chongqing, People’s Republic of China; 6 Chongqing International Institute for Immunology, 400030 Chongqing, People’s Republic of China; 7 Department of Nephrology, Chinese PLA General Hospital, Chinese PLA Institute of Nephrology, National Key Laboratory of Kidney Diseases, National Clinical Research Center for Kidney Diseases, 100853 Beijing, China

**Keywords:** Epigenetics in immune cells, Autoimmunity

## Abstract

Retinoid-related orphan receptor gamma t (RORγt) is the lineage-specific transcription factor for T helper 17 (Th17) cells. Our previous study demonstrated that STAT3 likely participates in the activation of RORCE2 (a novel enhancer of the RORγt gene) in Th17 cells. However, the detailed mechanism is still unclear. Here, we demonstrate that both STAT3 and SOX-5 mediate the enhancer activity of RORCE2 in vitro. Deletion of the STAT3 binding site (STAT3-BS) in RORCE2 impaired RORγt expression and Th17 differentiation, resulting in reduced severity of experimental autoimmune encephalomyelitis (EAE). Mechanistically, STAT3 and SOX-5 bind the RORCE2 region and recruit the chromatin remodeling factor BRG1 to remodel the nucleosomes positioned at this region. Collectively, our data suggest that STAT3 and SOX-5 mediate the differentiation of Th17 cells through the induction of BRG1-mediated chromatin remodeling of RORCE2 in Th17 cells.

## Abstract (web summary)

STAT3 and SOX-5 may mediate Th17 cell differentiation through the induction of BRG1-mediated chromatin remodeling of RORCE2.

## Introduction

T helper 17 (Th17) cells are a subset of CD4^+^ T-cells that are important for protecting the host against pathogens and for maintaining mucosal homeostasis^1–4^. However, Th17 cells also participate in the pathogenesis of a variety of autoimmune diseases, such as multiple sclerosis (MS) and psoriasis^5,6^. Initiation of the differentiation of Th17 cells from naïve CD4^+^ T-cells requires the presence of at least two cytokines: interleukin (IL)-6 and transforming growth factor-β (TGF- β)^7,8^. IL-6 acts through the signal transducer and activator of transcription 3 (STAT3) pathway^9,10^, but TGF-β is thought to act through a noncanonical Sma- and Mad-related protein (SMAD) pathway and relies on SMAD2 and tripartite motif-containing 33 (TRIM33)^11,12^. Retinoid-related orphan receptor gamma t (RORγt), which is encoded by the RORC gene, is a Th17-specific transcription factor (TF) and is required for the development and function of Th17 cells due to its functional activation of IL-17A and IL-17F gene expression^13^.

Cis-regulatory elements have important functions in controlling lineage-specific gene expression, such as RORγt expression, in Th17 cells^14–16^. In our previous studies, we identified a novel enhancer of RORγt in Th17 cells, termed RORCE2, whose deficiency downregulates RORγt expression and Th17 differentiation, leading to reduced susceptibility to experimental autoimmune encephalomyelitis (EAE)^17^. The enhancer activity of RORCE2 mainly depends on SRY-box transcription factor 5 (SOX-5) in Th17 cells^17^. SOX-5 belongs to the SOXD group, which does not have transactivation or transrepression domains^15,18^, and thus, its activity may be influenced by its partner proteins. We preliminarily confirmed that STAT3 participates in the activation of RORCE2 and that SOX-5 affects STAT3 recruitment to the RORCE2 region^17^. However, the detailed mechanism by which STAT3 and SOX-5 activate RORCE2 is still unclear.

In this study, we show that STAT3 and SOX-5 induce the activation of RORCE2 in Th17 cells, thus promoting RORγt expression and subsequent Th17 cell differentiation and EAE pathogenesis. Furthermore, the activation of RORCE2 depends on chromatin remodeling factor BRG1 (brahma-related gene 1; SMARCA4), which is recruited by STAT3 and SOX-5. In summary, our present study provides evidence for a novel mechanism underlying the regulation of RORCE2 activation mediated by STAT3 and SOX-5.

## Results

### STAT3 and SOX-5 activate RORCE2 in Th17 cells in vitro

**Fig. 1:**
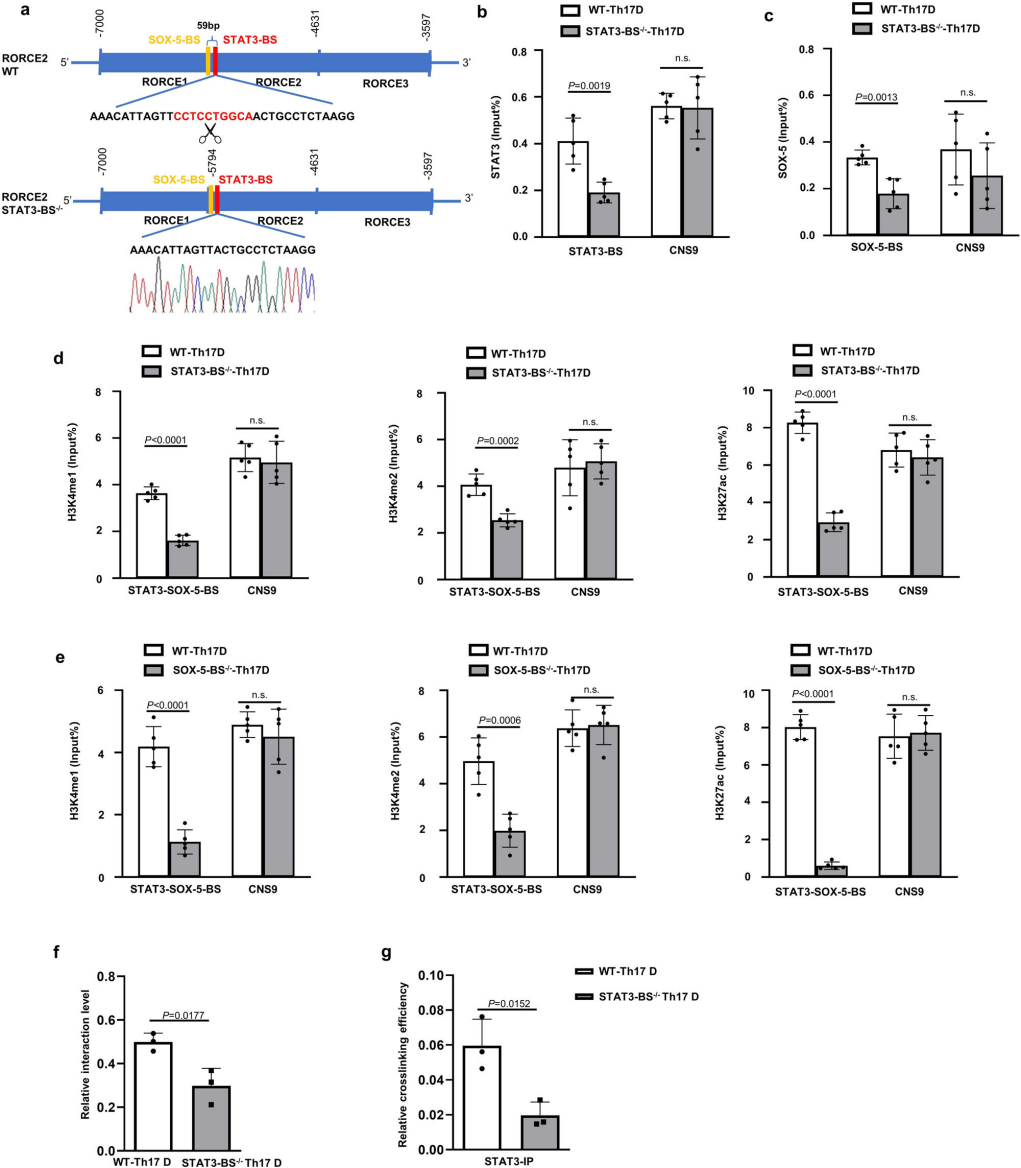
STAT3 and SOX-5 activate RORCE2 in Th17 cells. **a** The deletion of the STAT3-BS sequence in RORCE2 by CRISPR/Cas9 (top) and confirmation by sequencing (bottom). **b**–**d** ChIP‒qPCR assays were performed on WT and STAT3-BS^−/−^ Th17-polarized cells with the indicated antibodies. **e** ChIP‒qPCR was performed on WT and SOX-5-BS^−/−^ Th17-polarized cells with the indicated antibodies. STAT3-SOX-5-BS indicates the location covering the STAT3 binding site and SOX-5 binding site in RORCE2. CNS9 (+5802 to +7963 bp from the RORC TSS) indicates a cis-regulatory element distal to STAT3-BS in RORCE2 and can be bound by STAT3, as the negative control for ChIP‒qPCR in this study. **f**, **g** 3C-qPCR (f) and ChIP-loop assays (with an anti-STAT3 antibody) (**g**) were performed to evaluate the interaction between RORCE2 and the RORγt gene promoter in WT and STAT3-BS^−/−^ Th17-polarized cells. Means ± SEMs are shown, *n* = 5 biologically independent animals (**b**–**e**), *n* = 3 biologically independent animals (**f**, **g**). D = differentiation.

We first performed dual-luciferase experiments to confirm the role of the STAT3 binding site (BS) in RORCE2 enhancer activity^19,20^. The results showed that the deletion of STAT3-BS significantly impaired the enhancer activity of RORCE2, and the degree of the decrease was comparable to that caused by SOX-5-BS deletion (Supplementary Fig. 1). To further clarify the role of STAT3 in RORCE2 activity, we generated STAT3-BS-deficient (STAT3-BS^−/−^) mice using the CRISPR/Cas9 system (Fig. 1a). STAT3-BS deficiency led to a significant decrease in STAT3 binding to the RORCE2 region (Fig. 1b). Interestingly, deletion of STAT3-BS also decreased the enrichment of SOX-5 at the RORCE2 region (Fig. 1c). We then investigated the epigenetic modifications related to active enhancers in Th17-polarized cells from WT and STAT3-BS^−/−^ mice and found that the deletion of STAT3-BS significantly reduced H3K4me1, H3K4me2, and H3K27ac modifications in the RORCE2 region (Fig. 1d). Similar results were observed in Th17-polarized cells from SOX-5-BS^−/−^ mice (Fig. 1e). We further examined the RORCE2/RORγt promoter interaction by 3C-qPCR and ChIP-loop assays and found a decreased interaction after STAT3-BS deficiency in Th17-polarized cells (Fig. 1f, g). These in vitro results suggested that the binding of both STAT3 and SOX-5 at the RORCE2 region facilitated enhancer activity.

### STAT3-BS deficiency impairs Th17 cell differentiation

**Fig. 2:**
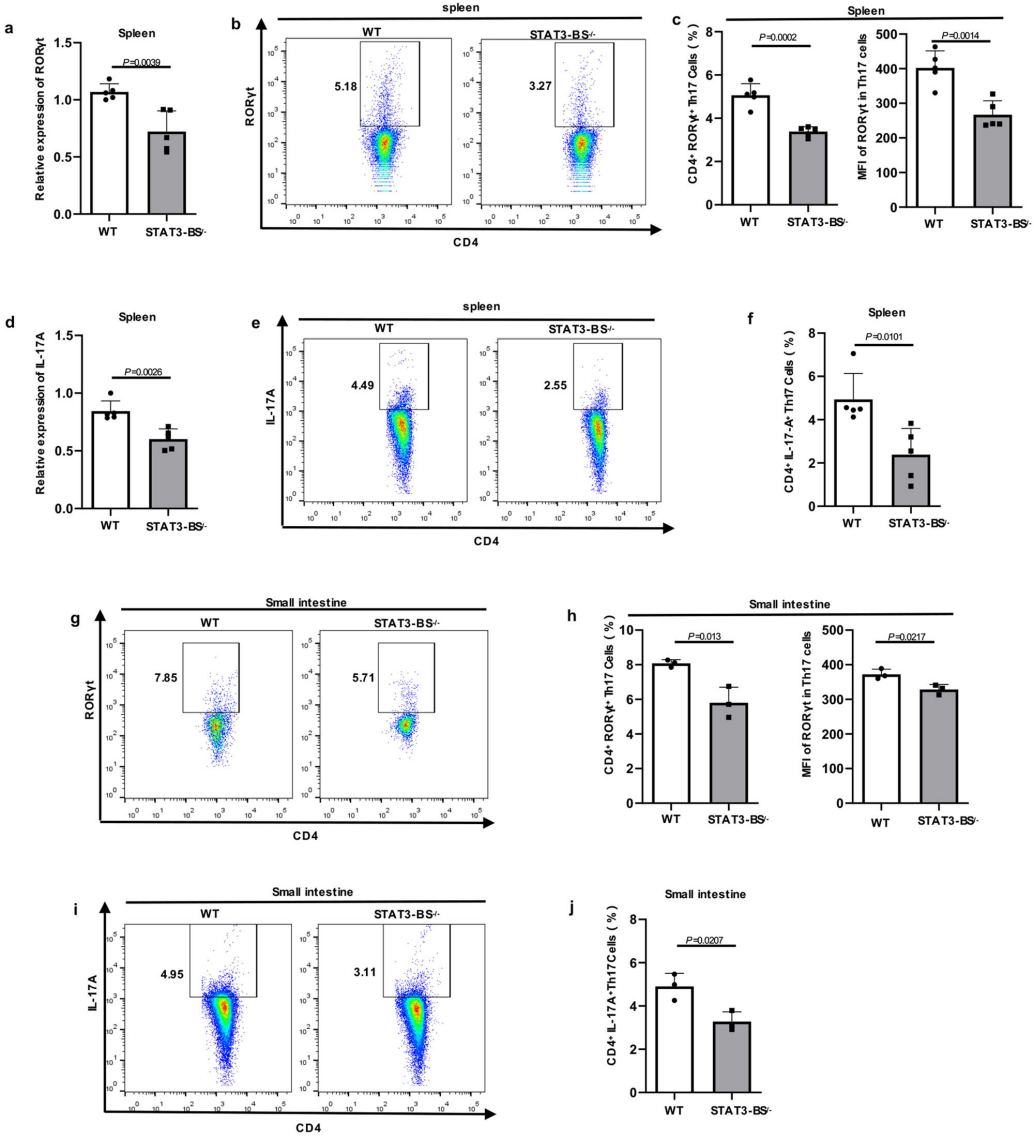
STAT3-BS deficiency of RORCE2 downregulates RORγt expression and Th17 cell differentiation in vivo. **a** The relative mRNA levels of *RORγt* in CD4^+^ T cells from the spleens of WT and STAT3-BS^−/−^ mice were quantified by RT‒qPCR. **b**, **c** RORγt^+^ Th17 cell frequency in splenic CD4^+^ T cells from WT and STAT3-BS^−/−^ mice and mean fluorescence intensity (MFI) of RORγt in RORγt^+^ Th17 cells were analyzed by flow cytometry (**b**) and statistically evaluated (**c**). **d** The relative mRNA levels of *IL-17A* in CD4^+^ T cells from the spleens of the WT and STAT3-BS^−/−^ mice. **e**, **f** IL-17A^+^ Th17 cell frequencies among splenic CD4^+^ T cells of the WT and STAT3-BS^−/−^ mice. **g**, **h** RORγt^+^ Th17 cell frequencies among CD4^+^ CD45^+^ Lin^+^ T cells were determined by flow cytometry in LPLs of the small intestine from the WT and STAT3-BS^−/−^ mice and RORγt MFI in Th17 cells. **i**, **j** IL-17A^+^ Th17 cell frequencies among CD4^+^ CD45^+^ Lin^+^ T cells from LPLs of the small intestine from the WT and STAT3-BS^−/−^ mice. Means ± SEMs are shown, *n* = 5 biologically independent animals (**a**, **c**, **d**, **f**), *n* = 3 biologically independent animals (**h**, **j**).

We then confirmed the role of STAT3-mediated RORCE2 activity in regulating RORγt expression and Th17 cell differentiation in vivo and in vitro. The results showed that STAT3-BS deficiency led to an approximately 30% reduction in the expression of RORγt (mRNA in splenic CD4^+^ T-cells and protein in splenic RORγt^+^ Th17 cells) (Fig. 2a, c). STAT3-BS^−/−^ mice exhibited a consistent decrease in RORγt^+^ Th17 cell frequencies among splenic CD4^+^ T-cells (Fig. 2b, c). As RORγt plays a crucial role in IL-17A induction in Th17 cells^15^, we further evaluated the change in IL-17A expression after STAT3-BS deletion. Our results revealed that the ablation of STAT3-BS in splenic CD4^+^ T cells led to a significant reduction in *IL-17A* expression and in IL-17A^+^ Th17 cell frequencies (Fig. 2d–f). STAT3-BS^−/−^ mice showed similar decreases in Th17 cells frequencies among lamina propria lymphocytes (LPLs) in the small intestine (Fig. 2g–j). However, there was no change in the expression of Th1 or Th2 signature genes or in the frequency of Th1 or Th2 cells in splenic CD4^+^ T-cells after STAT3-BS deficiency (Supplementary Fig. 2a–d and 3a–d). In addition, given the similarities between Th17 cells and group 3 innate lymphoid cells (ILC3s), we investigated the influence of STAT3-mediated RORCE2 activity on ILC3s. In contrast to those of Th17 cells in the LPLs of STAT3-BS^−/−^ mice, the ILC3 frequencies and the RORγt mean fluorescence intensity (MFI) in ILC3s were not significantly influenced (Supplementary Fig. 4a, b).

**Fig. 3:**
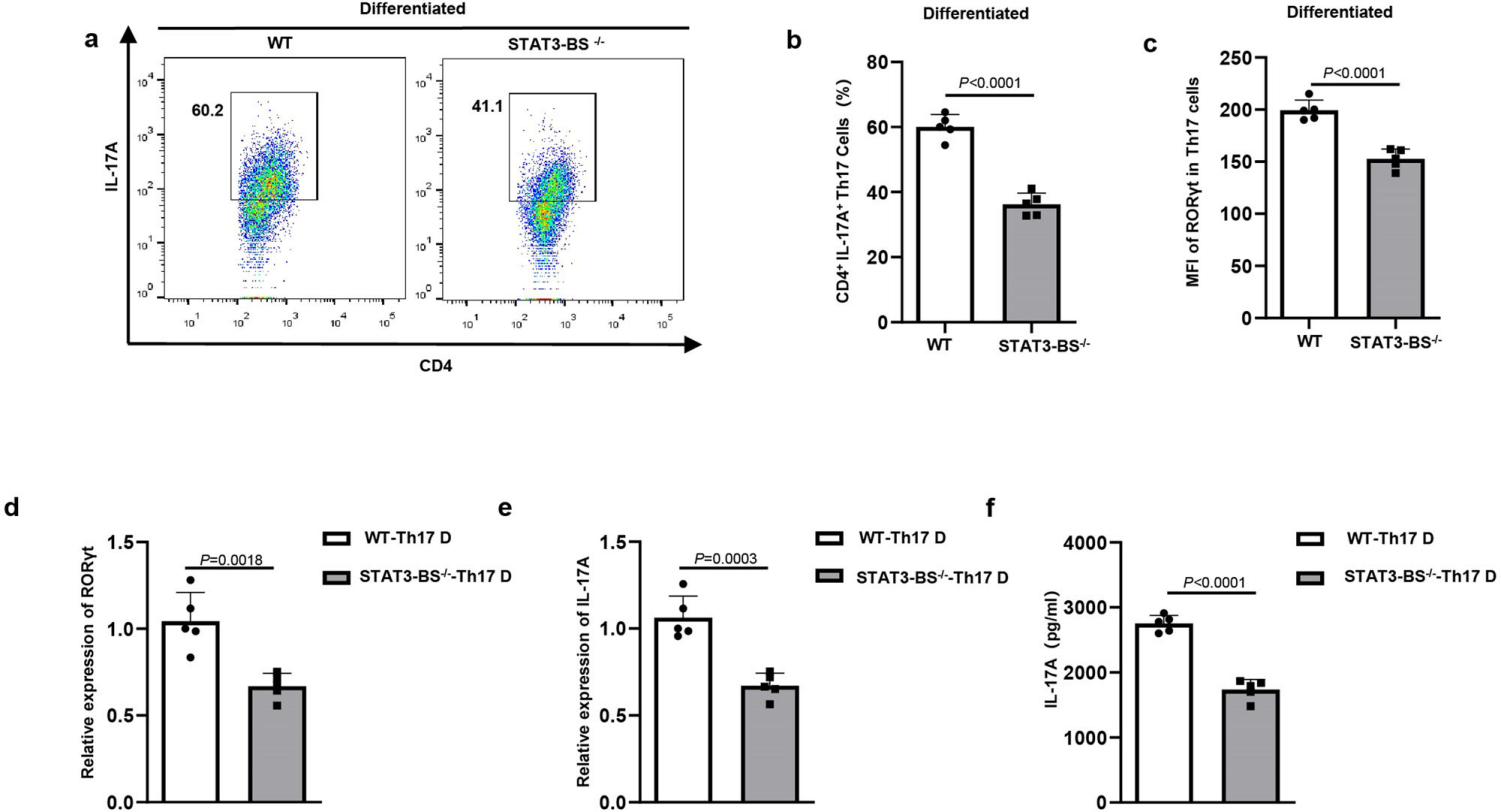
STAT3-BS deficiency of RORCE2 results in impaired Th17 cell polarization in vitro. Naïve CD4^+^ T cells from the spleens of WT or STAT3-BS^−/−^ mice were cultured in Th17-polarizing conditions for 3 days. **a**, **b** Th17 cell frequencies among CD4^+^ T cells. **c** MFI of RORγt in RORγt^+^ Th17 cells. **d**, **e** Relative mRNA levels of *RORγt* (**d**) and *IL-17A* (**e**) in Th17-polarized cells. **f** An ELISA was used to detect IL-17A production in culture supernatants. Means ± SEMs are shown, *n* = 5 biologically independent animals (**b**–**f**). D = differentiation.

Naïve CD4^+^ T cells of STAT3-BS^−/−^ mice and WT mice were polarized into Th17 cells to confirm the effect of STAT3-BS deficiency in RORCE2 on Th17 cell differentiation in vitro. Disruption of STAT3-BS significantly reduced the Th17 cell frequencies (Fig. 3a, b). Furthermore, significant reductions in the expression of RORγt (the mRNA expression level in Th17-polarized cells and the RORγt MFI in RORγt^+^ Th17 cells) and in *IL-17A* mRNA expression and secretion were observed in Th17-polarized cells from STAT3-BS^−/−^ mice compared to those from WT mice (Fig. 3c–f). Notably, deletion of STAT3-BS did not impair the frequencies of Th1 or Th2 cell differentiation, mRNA expression of Th1 or Th2 signature genes or cytokine production in Th1- or Th2-polarized cells (Supplementary Fig. 5a–e and Fig. 6a–e). Collectively, our data suggest that STAT3-mediated RORCE2 activity plays an important role in Th17 cell differentiation.

### The severity of EAE is alleviated in STAT3-BS^−/−^ mice compared to WT mice

**Fig. 4:**
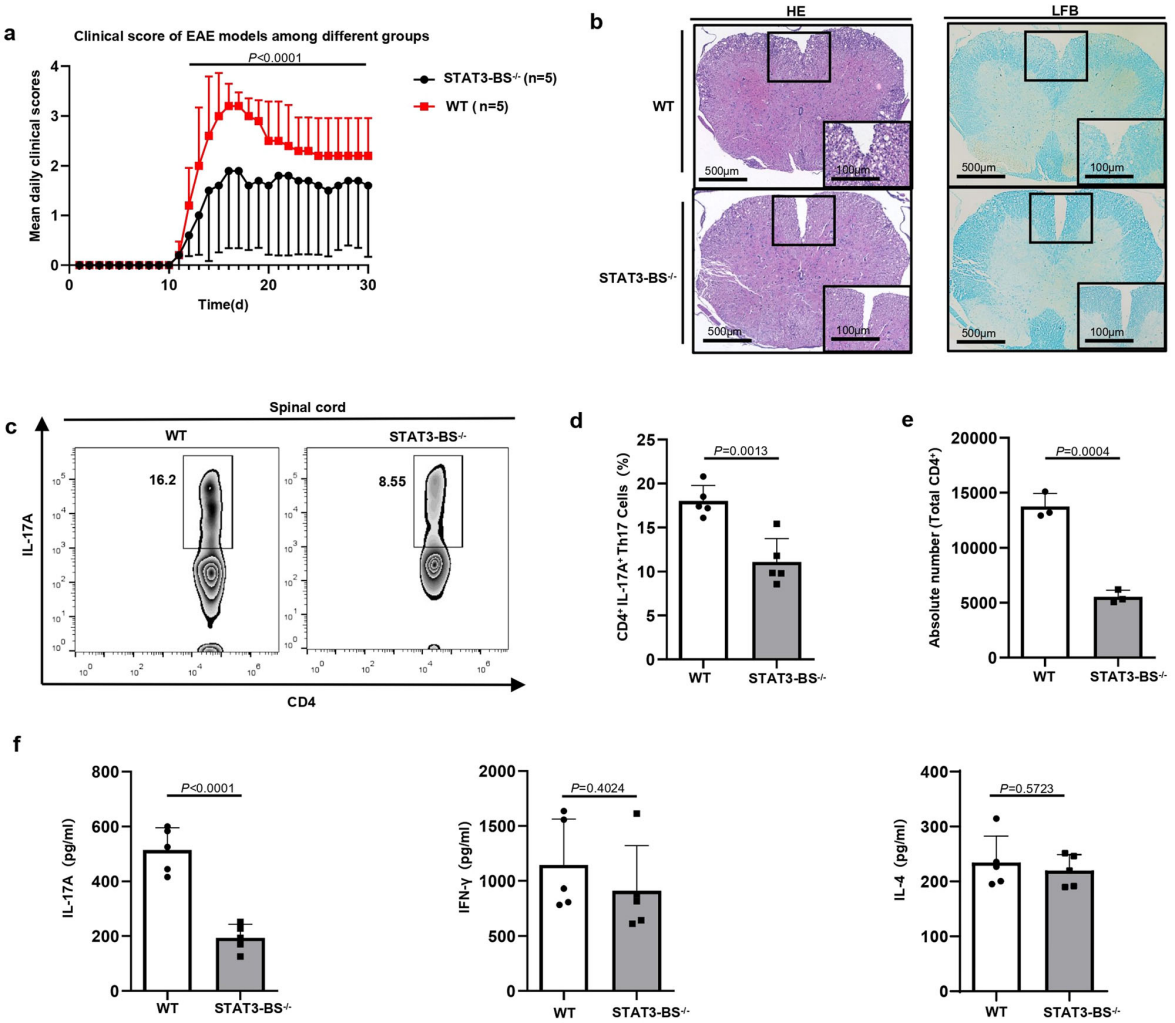
STAT3-BS deficiency in RORCE2 alleviates the severity of EAE. **a** The mean daily clinical scores of EAE for WT and STAT3-BS^−/−^ mice are shown. **b** Representative staining image of hematoxylin-eosin (HE) and luxol fast blue (LFB) in the spinal cord sections at 30 days after immunization. Scale bars, 500 or 100 µm. **c**–**e** Flow cytometry analysis of mononuclear cells from the spinal cord of EAE-induced WT and STAT3-BS^−/−^ mice at 30 days after immunization. Th17 cell frequencies among CD4^+^ T cells (**c**, **d**). The numbers of infiltrating CD4^+^ T cells in the spinal cord (**e**). **f** Inguinal lymph node cells were extracted at day 8 after immunization and restimulation with MOG peptide for 3 days. IL-17A, IFNγ, and IL-4 production were examined by ELISA. Means ± SEMs are shown, *n* = 5 biologically independent animals (**d**, **f**), *n* = 3 biologically independent animals (**e**).

To evaluate the functional importance of the STAT3-mediated activity of RORCE2 in the development of autoimmune diseases, EAE was induced in age- and sex-matched STAT3-BS^−/−^ and WT mice. Although both WT and STAT3-BS^−/−^ mice started to develop EAE disease symptoms approximately 10 days after immunization, the disease severity was remarkably lower in STAT3-BS^−/−^ mice than in WT mice (Fig. 4a). Histological analysis of the spinal cord demonstrated reductions in the infiltration of inflammatory cells and demyelization in STAT3-BS^−/−^ mice compared to WT mice (Fig. 4b). In the spinal cords of the STAT3-BS^−/−^ mice, there was a consistent notable decrease in the Th17 cell frequencies and in the absolute numbers of infiltrating CD4^+^ T cells (Fig. 4c–e), reflecting the lesser disease severity in the STAT3-BS^−/−^ mice. In addition, the percentages of Th1 and Th2 cells in the spinal cords of the STAT3-BS^−/−^ mice were also reduced compared to those of WT mice (Supplementary Fig. 7a–d). To verify that the alleviated severity of EAE in STAT3-BS^−/−^ mice was Th17-related, WT and STAT3-BS^−/−^ mice were immunized with MOG_35–55_ peptide. Draining lymph nodes were extracted on day 8 after immunization and subsequently restimulated with MOG for 3 days. The IL-17A concentration was decreased in STAT3-BS^−/−^ mice, while IFNγ and IL-4 production was unchanged (Fig. 4f). These data demonstrate the significant role of STAT3-mediated RORCE2 activity in Th17-related autoimmune disease.

It is well known that IL-17-producing γδT cells (γδT17 cells) can exacerbate EAE. STAT3 is critical for γδT17 cell responses under inflammatory conditions^21^. In addition, SOX-13, a member of the SOX-D family (including SOX-5), is essential for the development of γδT17 cells^22^. We then determined the frequencies of γδT17 cells in the lymph nodes of naïve STAT3-BS^−/−^ and WT mice and the draining lymph nodes of STAT3-BS^−/−^ and WT mice upon EAE induction. The frequencies of TCRγδ^+^ IL-17A^+^ γδT17 cells in the lymph nodes and draining lymph nodes of STAT3-BS^−/−^ mice were decreased compared to those in WT mice (Supplementary Fig. 8a–d). These data demonstrate that STAT3-mediated RORCE2 activity also affects γδT17 cell frequencies both under physiological conditions and upon EAE induction.

### STAT3 and SOX-5 activate RORCE2 by recruiting the chromatin remodeling factor BRG1 in Th17 cells

**Fig. 5:**
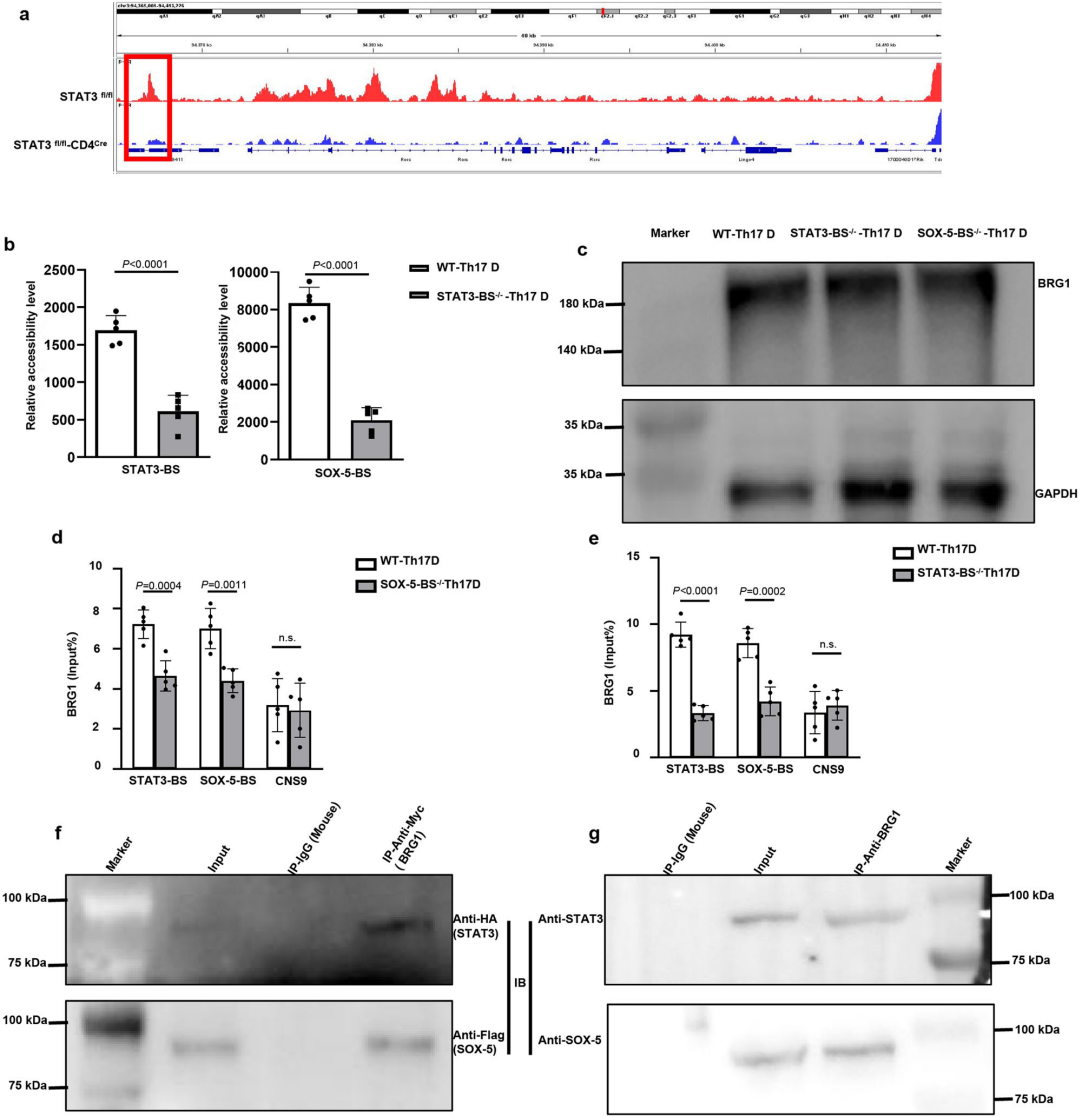
The binding of STAT3 and SOX-5 on RORCE2 is essential for the recruitment of BRG1. **a** ATAC-seq data at the RORC locus in STAT3^fl/fl^ Th17 cells versus STAT3^fl/fl^ -CD4^Cre^ Th17 cells. **b** A chromatin accessibility assay was performed on SOX-5-BS and STAT3-BS in RORCE2 in the WT and STAT3-BS^−/−^ Th17-polarized cells. **c** The expression of BRG1 and GAPDH proteins in the WT, STAT3-BS^−/−^ and SOX-5-BS^−/−^Th17-polarized cells was detected by Western blotting. **d**, **e** ChIP‒qPCR assays of BRG1 were performed in the WT, STAT3-BS^−/−^ and SOX-5-BS^−/−^Th17-polarized cells. CNS9 (+5802 to +7963 bp from the RORC TSS) indicates a cis-regulatory element distal to STAT3-BS in RORCE2 and can be bound by STAT3, as the negative control for ChIP‒qPCR in this study. **f** Transfected HeLa cells were subjected to IP with the indicated antibody. Input and IP proteins were then subjected to IB with the indicated antibody. **g** Whole-cell lysates from Th17 cells were subjected to IP with an anti-BRG1 antibody or control mouse IgG and to IB with an anti-STAT3 or anti-SOX-5 antibody. Input proteins (input) were also subjected to IB with an anti-STAT3 or anti-SOX-5 antibody. Means ± SEMs are shown, *n* = 5 biologically independent animals (**b**, **d**, **e**). D = differentiation.

To elucidate the mechanisms underlying SOX-5- and STAT3-mediated RORCE2 activation, we re-analyzed previously published assay for transposase-accessible chromatin with high throughput sequencing (ATAC-seq) data for the RORC locus in STAT3^fl/fl^ and STAT3^fl/fl^ CD4^Cre^ Th17 cells^16^ and found that chromatin accessibility in the RORCE2 region was significantly decreased in STAT3^fl/fl^-CD4^Cre^ Th17 cells (Fig. 5a). Consistent with the ATAC-seq results, our results further showed that the deletion of STAT3-BS also reduced chromatin accessibility in the RORCE2 region, suggesting that STAT3 might contribute to chromatin remodeling of the RORCE2 region (Fig. 5b). Similar results were observed in SOX-5-BS^−/−^ mice (Supplementary Fig. 9a, b). The structural changes in chromatin are catalyzed by chromatin remodeling enzymes^23^. Previous studies have shown that the SWI/SNF remodeling ATPase BRG1 is important for the development, differentiation, and function of various cell types, including T cells and ILC3s^23–26^. We thus analyzed the role of BRG1 in Th17 cell differentiation. High protein levels of BRG1 were detected by Western blot in STAT3-BS^−/−^, SOX-5-BS^−/−^ and WT Th17-polarized cells (Fig. 5c). We also measured BRG1 protein expression in Th1-, Th2- and Treg-polarized cells and found comparable levels in these cells compared to Th17-polarized cells (Supplementary Fig. 10a). We further explored the role of BRG1 in SOX-5- and STAT3-mediated RORCE2 enhancer activity. Interestingly, the ChIP‒qPCR results showed that BRG1 binding to the RORCE2 region was significantly impaired in SOX-5-BS^−/−^ and STAT3-BS^−/−^ Th17-polarized cells (Fig. 5d, e). Furthermore, BRG1 is unable to bind chromatin alone but is recruited by transcription factors, which can displace, unfold, and slide nucleosomes^27,28^. The co-immunoprecipitation (Co-IP) results demonstrated that both SOX-5 and STAT3 interacted with BRG1 (Fig. 5f, g). These results suggest that BRG1 is recruited by SOX-5 and STAT3 to bind RORCE2 region in Th17 cells.

## Discussion

In this study, we elucidated the mechanism by which STAT3 and SOX-5 mediate RORCE2 activation in Th17 cells. Deletion of STAT3-BS in RORCE2 markedly reduced the enhancer activity of RORCE2, resulting in decreased RORγt expression, Th17 differentiation, and EAE severity. Finally, we demonstrated that both STAT3 and SOX-5 can recruit the chromatin-remodeling factor BRG1 to bind at the RORCE2 region, contributing to chromatin accessibility and further activating the enhancer RORCE2 in Th17 cells.

Studies have shown that the SOXD family genes, whose transcriptional potential is fulfilled by specific partner proteins, lack transactivation or transrepression domains^15,18^. SOX-5 has three functional domains, including one HMG box DNA-binding domain and two coiled-coil domains. A previous study showed that SOX-5 interacts with c-Maf via its HMG domain and the DNA-binding domain of c-Maf, thus activating the RORγt promoter in CD4^+^ T cells^15^. We previously demonstrated that STAT3 interacted with SOX-5^17^. In this study, we showed that both STAT3 and SOX-5 recruited the chromatin-remodeling factor BRG1 to activate RORCE2 in Th17 cells. STAT3 consists of six domains, including the DNA-binding domain^29^. Therefore, SOX-5 probably associates with STAT3 via the HMG domain of SOX-5 and the DNA-binding domain of STAT3, but further experimental evidence needs to be provided.

In contrast to previous studies that mainly focused on the knockout or knockdown of TF genes to investigate enhancer-promoter looping and their functions^30–33^, we used CRISPR‒Cas9 technology to specifically knock out STAT3-BS in RORCE2. More direct and more accurate evidence will be provided by this approach to confirm the importance of TF binding in the enhancer, because knockout of a specific TF may result in unpredictable effects on cell biology based on the multiple targets of the TF^34^; this highlights the significance of our approach.

This study demonstrated that BRG1 facilitated RORCE2 activation by ATP-dependent chromatin-remodeling activity, resulting in RORγt expression and Th17 differentiation. Previous studies have shown that BRG1 can promote Th1/Th2 differentiation by remodeling the chromatin structure of the cytokine genes: IFN-γ and IL-4^23,25^. The remodeling activity of BRG1 reacts with the RORγt enhancer RORCE2 to facilitate the function of Th17 cells, which outweighs the importance of BRG1 in regulating the differentiation and gene expression of CD4^+^ T cells.

In summary, we detailed the exhaustive regulatory mechanism underlying RORCE2 activation in Th17 cells. Our results showed that STAT3 and SOX-5 bind to RORCE2 region and recruit BRG1 to remodel nucleosomes, thus activating this enhancer, which was essential for the interaction between RORCE2 and the RORγt gene promoter and subsequently promoted RORγt expression and Th17 cell differentiation.

## Methods

### Generation of STAT3-BS-deficient mice

STAT3-BS^−/−^ mice were generated by the CRISPR/Cas9 system^35,36^. Briefly, sgRNAs were used to target STAT3-BS (chr3:94,367,026-94,367,035) (sequences of sgRNAs: 5′-CCTTAGAGGCAGTTGCCAGGAGG-3′ and 5′-TGACCTTTATAAGCCACAAGAGG-3′). Zygotes were co-injected with sgRNA and Cas9 mRNA and were then implanted into surrogate mice. The STAT3-BS-deficient mice were generated by Beijing Biocytogen Co., Ltd. The primers used for genotyping were 5′-GAGTCATGTCACCTAGTTTTC-TTCT-3′ and 5′-CTTGCTCCAGTTGTCCACCA-3′. Sequence analysis was performed on the PCR products.

All the experimental/control mice were maintained on a C57BL/6 background and were kept in standard cages (4–5 mice per cage) under specific pathogen–free conditions with food and water at stable room temperature and a 12/12-h light/dark cycle. Mice aged 8 to 12 weeks were used for experiments without sex preference. We have received ethical approval for animal use from the Institutional Animal Care and Use Committees of the Third Military Medical University.

### Dual luciferase reporter assays

The dual-luciferase reporter assays were conducted as described previously^17^. Briefly, all deletion mutants of RORCE2 (RORCE2 without STAT3-BS, SOX-5-BS or STAT3-SOX-5-BS) were constructed by Qsingke Biotechnology Corp. (Shanghai, China) and cloned upstream of the RORγt promoter in pGL3 vectors. 293 T cells were then transfected with the indicated plasmids together with pRL-TK (as a normalization of the transfection efficiency) by Lipofectamine 3000 (Invitrogen, L3000015) and cultured for 24 h. Following the 24-h transfection, the 293T cells were stimulated by PMA (50 ng/ml, Sigma‒Aldrich, P8139) and ionomycin (1 µg/ml, Sigma‒Aldrich, 13909) for 4-6 h, subsequently lysed, and tested using a dual-luciferase reporter system (Promega, E1910). The firefly and Renilla luciferase activities were measured in turn, and the ratio (firefly luciferase activity: Renilla luciferase activity) is shown as the relative luciferase activity. All experiments were repeated five times.

### Isolation of splenic CD4^+^ T cell

The spleens were collected from the indicated mice (8 to 12 weeks of age) and pressed through a 40-µm cell strainer. Spleen cells were prepared by lysing erythrocytes with red blood cell lysis buffer (TIANGEN, RT122), and splenic CD4^+^ T cells were sorted by an EasySep™ mouse CD4^+^ T cell isolation kit (STEMCELL Technologies, 19852).

### Isolation of splenic naïve CD4^+^ T cell

The spleens were collected from the indicated mice (8 to 12 weeks of age) and pressed through a 40-µm cell strainer. Spleen cells were prepared by lysing erythrocytes with red blood cell lysis buffer (TIANGEN, RT122), and splenic naïve CD4^+^ T cells were sorted by an EasySep™ mouse naïve CD4^+^ T cell isolation kit (STEMCELL Technologies, 19765).

### Isolation of lymph node cells

The lymph nodes were collected from the indicated mice (8 to 12 weeks of age) and pressed through a 40-µm cell strainer. Lymph node cells were prepared by lysing erythrocytes with red blood cell lysis buffer (TIANGEN, RT122).

### ChIP‒qPCR

ChIP was performed according to the instructions of the ChIP assay kit (Beyotime, P2078). Antibodies for ChIP included anti-SOX-5 (Abcam, ab94396, 1:150), anti-STAT3 (Cell Signaling Technology, 9132, 1:150), anti-H3K27Ac (Abcam, ab4729, 1:150), anti-H3K4me1 (Abcam, ab8895, 1:150), anti-H3K4me2 (Abcam, ab7766, 1:150), and anti-BRG1 (AiFang Biological, AF300790, 1:150). Extracted DNA was quantified by real-time PCR with TB Green Premix Ex Taq II (TaKaRa, RR820A) and normalized to the input. The ChIP‒qPCR primers are listed in Supplementary Table 1.

### RT‒qPCR

Total RNA was extracted by TRIzol (Invitrogen) and reverse-transcribed to cDNA (TaKaRa, RR047A). Relative gene expression was analyzed with TB Green Premix Ex Taq II (TaKaRa, RR820A). The 2^-ΔΔCt^ method was used for quantification of the relative mRNA expression, and *GAPDH* was used as the internal control. The real-time PCR primers are listed in Supplementary Table 2.

### Differentiation of CD4^+^ T cells in vitro

Differentiation of CD4^+^ T cells was performed according to our previous study^17^. In brief, splenic naïve CD4^+^ T cells were sorted from the indicated mice (8 to 12 weeks of age) by an EasySep™ mouse naïve CD4^+^ T cell isolation kit (STEMCELL Technologies, 19765). Th17, Th1 and Th2 polarizations were then conducted following the instructions of the CellXVivo mouse Th17 cell differentiation kit (R&D Systems, CDK017), the CellXVivo mouse Th1 cell differentiation kit (R&D Systems, CDK018) and the CellXVivo mouse Th2 cell differentiation kit (R&D Systems, CDK019), respectively.

### Flow cytometry

For surface staining, cells were incubated with the indicated surface antibodies for 30 min at 4 °C. For intracellular staining of cytokines, cells were stimulated with GolgiPlug (1 μl/ml, BD Biosciences, 51-2301K7), protein transport inhibitor Golgi-Stop (3 μl/ml, BD Bioscience, 51-2092KZ), PMA (50 ng/ml, Sigma‒Aldrich, P8139) and ionomycin (1 µg/ml, Sigma‒Aldrich, 13909) for 4-6 h. After surface antibody staining, cells were fixed, permeabilized with a Fixation/Permeabilization Solution Kit (BD Biosciences, 554722), and subsequently stained with appropriate antibodies for 1 h at 4 °C. For intracellular staining of TFs, cells were fixed and permeabilized with the FOXP3/Transcription Factor Buffer Set (BioLegend, 421403). Data were collected with a BD FACSCantoII (BD Biosciences) and analyzed with FlowJo 10.0.7 software. The gating strategies used for frequency analysis were shown in Supplementary Fig. 12.

The mAbs used for Th1, Th2, and Th17 cell staining were as follows: anti-CD4 (BioLegend, 100540), anti-IFNγ (eBioscience, 17-7311-82-82), anti-IL-4 (eBioscience, 17-7041-82), anti-RORγt (eBioscienc, 17-6988-82, 12-6981-82), and anti-IL-17A (BioLegend, 506904).

The mAbs used for LPL staining were anti-lineage cocktail (including anti-CD3 (Invitrogen, 17-0032-80), anti-CD19 (BioLegend, 25-0451-82), anti-CD8 (eBioscience, 17-0081-82), and anti-Gr1 (BioLegend, 108412) antibodies) and anti-CD45 (Invitrogen, 48-1271-82), anti-CD127 (Invitrogen), anti-CD4 (BioLegend, 100540), anti-RORγt (eBioscienc, 17-6988-82, 12-6981-82) and anti-IL-17A (BioLegend, 506904) antibodies.

The mAbs used for spinal cord mononuclear cell staining were anti-CD45 (Invitrogen, 48-1271-82), anti-CD3 (Invitrogen, 17-0032-80), anti-CD4 (BioLegend, 100540), anti-IFNγ (eBioscience, 17-7311-82-82), anti-IL-4 (eBioscience, 17-7041-82), and anti-IL-17A (BioLegend, 17-7041-82) antibodies. The concentrations of flow cytometry antibodies: 1:200.

### ELISA

The concentrations of secreted IL-17A, IFNγ, and IL-4 in cell culture supernatants were measured following the instructions of the Mouse IL-17A Precoated ELISA Kit (Dakewe, 1211702), Mouse IFN-γ Precoated ELISA Kit (Dakewe, 1210002) and Mouse IL-4 Precoated ELISA Kit (Dakewe, 1210402), respectively.

### Isolation and analysis of lamina propria lymphocytes (LPLs) in the small intestine

The small intestines were removed, cleared, dissected longitudinally, and cut into 1 cm pieces, followed by washing with cold PBS. The pieces were then incubated with predigestion solution (PBS containing 5 mM EDTA, 1 mM DTT, 10 mM HEPES and 5% FBS) twice for 20 min at 37 °C while shaking at 250 rpm. To remove the epithelial cells, the tissues were vortexed and filtered through a 100-µm cell strainer, washed, minced into small pieces, and digested with the Lamina Propria Dissociation Kit (Miltenyi Biotec, 130-097-410). The LPLs were isolated through gradient centrifugation by 40%/80% Percoll (GE Healthcare)^37^ and subjected to flow cytometric analysis.

### Induction of EAE

On day 0, age- and sex-matched WT and STAT3-BS-deficient mice were immunized with MOG_33–35_ peptide (200 µg/mouse, CHINESE PEPTIDE, MOG001) emulsified in complete Freund’s adjuvant (CFA, Sigma‒Aldrich, F5506) containing heat-inactivated *Mycobacterium tuberculosis* (5 mg/ml, BD Bioscience, 231141). In addition, 200 ng pertussis toxin (PTX, Millipore, 516562) was intraperitoneally injected into the mice 2 and 26 h after MOG immunization. The disease severity in immunized mice was scored daily as follows: healthy, 0; limp tail, 1; weakness in hind limbs, 2; complete paralysis of hind limbs, 3; complete hind limb and partial front limb paralysis, 4; and moribund state, 5^37^.

### Spinal cord mononuclear cell isolation

Spinal cord mononuclear cell isolation was performed according to our previous study^17^. Briefly, the spinal cord was cut into small pieces and then digested with the Neural Tissue Dissociation Kit (Miltenyi Biotec, 130-092-628). Mononuclear cells were isolated through gradient centrifugation on a sucrose solution (0.9 M) and subjected to flow cytometric analysis.

### Immunoblotting (IB) and coimmunoprecipitation (Co-IP) assays

IB and Co-IP assays were performed as previously described^17^. The following vectors were used for overexpression in HeLa cells: pcDNA3.1-BRG1 Myc, which expressed Myc-tagged BRG1; pcDNA3.1-SOX-5 Flag, which expressed Flag-tagged SOX-5; and pcDNA3.1-STAT3 HA, which expressed HA-tagged STAT3. The antibodies used for IP or IB included anti-Myc antibody (Abcam, ab32), anti-HA (Abcam, ab18181) and anti-Flag (Sigma‒Aldrich, F7425). The concentrations of Co-IP antibodies: 1:200, the concentrations of IB antibodies: 1:1000. Retrovirus-mediated overexpression of SOX-5 and STAT3 was performed by the RetroNectin-bound virus infection method as previously described^17^.

### ATAC-seq data processing

The ATAC-seq data of STAT3^fl/fl^ Th17 cells and STAT3^fl/fl^-CD4^Cre^ Th17 cells shown in Fig. 5a were obtained from GenBank: GSE153442 (Th17 STAT3-WT: GSM4644335, Th17 STAT3-KO: GSM4644336)^16^, and the data were processed as described previously^16^.

### Chromatin accessibility assay

Analysis of chromatin accessibility was tested by the EpiQuik™ Chromatin Accessibility Assay Kit (Epigentek, P-1047-48) according to the manufacturer’s instructions. In brief, cells were collected and washed with PBS, resuspended in 1X Lysis Buffer (400 µl/1 * 10^6^, 200 µl of cell suspension as a sample and another 200 µl as a no-Nse control) and incubated on ice for 10 min. After cell lysis, the chromatin was treated with a nuclease mix (Nse), and DNA was then isolated and amplified with real-time PCR for region-specific analysis of chromatin accessibility. Fold enrichment (FE) was calculated using the ratio of amplification efficiency of the Nse-treated DNA sample over that of the No-Nse control sample. FE% = 2 ^(Nse CT – no-Nse CT)^ × 100%. FE% of the untreated samples > 1600% compared to the Nse-treated samples, which indicates that the gene region is in the opened chromatin, while FE% of the untreated samples < 400% compared to the Nse-treated samples represents the gene region in closed chromatin.

### Statistics and reproducibility

GraphPad Prism 8.0 was used for experimental statistical analysis. The results are presented as Means ± SEMs. For two groups, statistical analysis was performed using the unpaired Student’s *t* test (two-tailed). For clinical score of EAE model, two-way analysis of variance (ANOVA) was used. The exact *P* values are shown in the figures (unless *P* < 0.0001). *P* > 0.05 was considered nonsignificant (n.s.).

### Reporting summary

Further information on research design is available in the Nature Portfolio Reporting Summary linked to this article.

### Supplementary information

Supplementary information

Description of Additional Supplementary Files

Supplementary Data 1

Reporting Summary

## Data Availability

The ATAC-seq data of STAT3^fl/fl^ Th17 cells and STAT3^fl/fl^-CD4^Cre^ Th17 cells shown in Fig. 5a were obtained from GenBank: GSE153442 (Th17 STAT3-WT: GSM4644335, Th17 STAT3-KO: GSM4644336)^16^. Uncropped and unedited blot/gel images are provided in Supplementary Fig. 11. Gating strategies used for frequency analysis are provided in Supplementary Fig. 12. The source data for the figures in this study are provided in Supplementary Data 1.
