# Supplementary material for: STAT3 and SOX-5 induce BRG1-mediated chromatin remodeling of RORCE2 in Th17 cells
Source: Commun Biol. 2024 Jan 3;7:10. doi: 10.1038/s42003-023-05735-9 (PMC10764326; doi:10.1038/s42003-023-05735-9)
Supplement: Supplementary file 1 — Supplementary information [file 42003_2023_5735_MOESM1_ESM.pdf]

Supplementary information

STAT3 and SOX-5 induce BRG1-mediated chromatin remodeling of RORCE2 in Th17 cells

Xian Wang[1,2†], Chao Han[1†], Di Yang[1], Jian Zhou[1], Hui Dong[1], Zhiyuan Wei[3], Shuai Xu[4], Chen Xu[1], Yiwei Zhang[1], Yi Sun[3], Bing Ni[5], Sheng Guo[1], Jingbo Zhang[4], Tingting Zhao[6], Xiangmei Chen[7], Yuzhang Wu[1,6*], Yi Tian[1*].

Affiliations

1. Institute of Immunology, Third Military Medical University (Army Medical University), Chongqing 400038, People's Republic of China.
2. Department of Immunology, Medical College of Qingdao University, Qingdao, Shandong 266071, People's Republic of China.
3. The First Affiliated Hospital, Third Military Medical University (Army Medical University), Chongqing 400038, People's Republic of China.
4. The Second Affiliated Hospital, Third Military Medical University (Army Medical University), Chongqing 400037, People's Republic of China.
5. Department of Pathophysiology, Third Military Medical University (Army Medical University), Chongqing 400038, People's Republic of China.
6. Chongqing International Institute for Immunology, Chongqing 400030, People's Republic of China.
7. Department of Nephrology, Chinese PLA General Hospital, Chinese PLA Institute of Nephrology, National Key Laboratory of Kidney Diseases, National Clinical Research Center for Kidney Diseases, Beijing 100853, China.

[†]These authors contributed equally to this work.

*Corresponding author. Email: tianyi@tmmu.edu.cn; wuyuzhang@tmmu.edu.cn or wuyuzhang@iiicq.vip.

Supplementary Fig. 1 Deletion of STAT3-BS significantly impairs the enhancer activity of RORCE2.

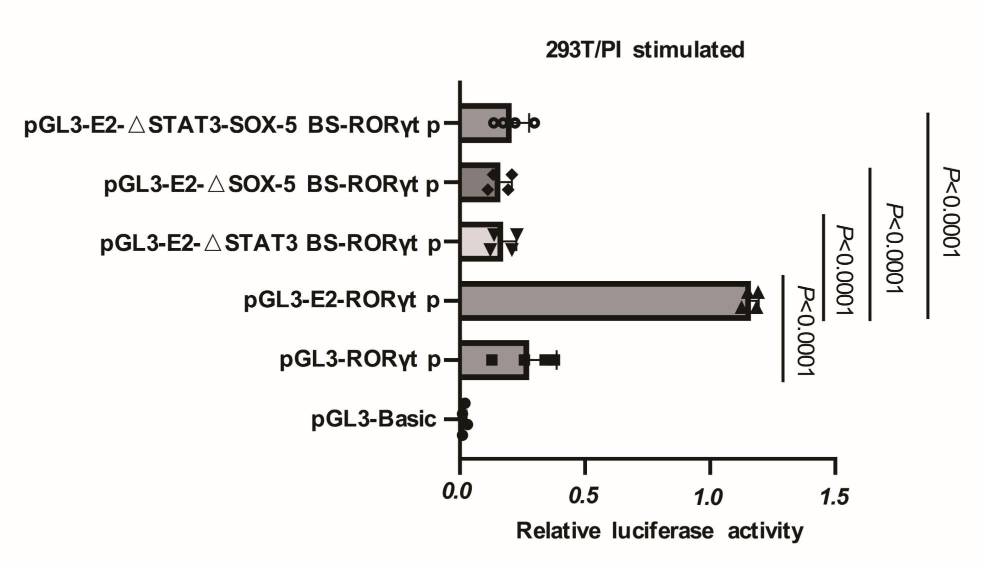

Dual-luciferase reporter assays of the indicated reporter constructs in 293T cells. E2-ΔSTAT3 BS = RORCE2 with STAT3-BS deletion; E2-ΔSOX-5 BS = RORCE2 with SOX-5-BS deletion; E2-ΔSTAT3-SOX-5 BS = RORCE2 with STAT3-BS and SOX-5-BS deletion. Means ± SEMs are shown, n = 5 independent experiments.

Supplementary Fig. 2 STAT3-BS deficiency has no effect on Th1 cells in vivo.

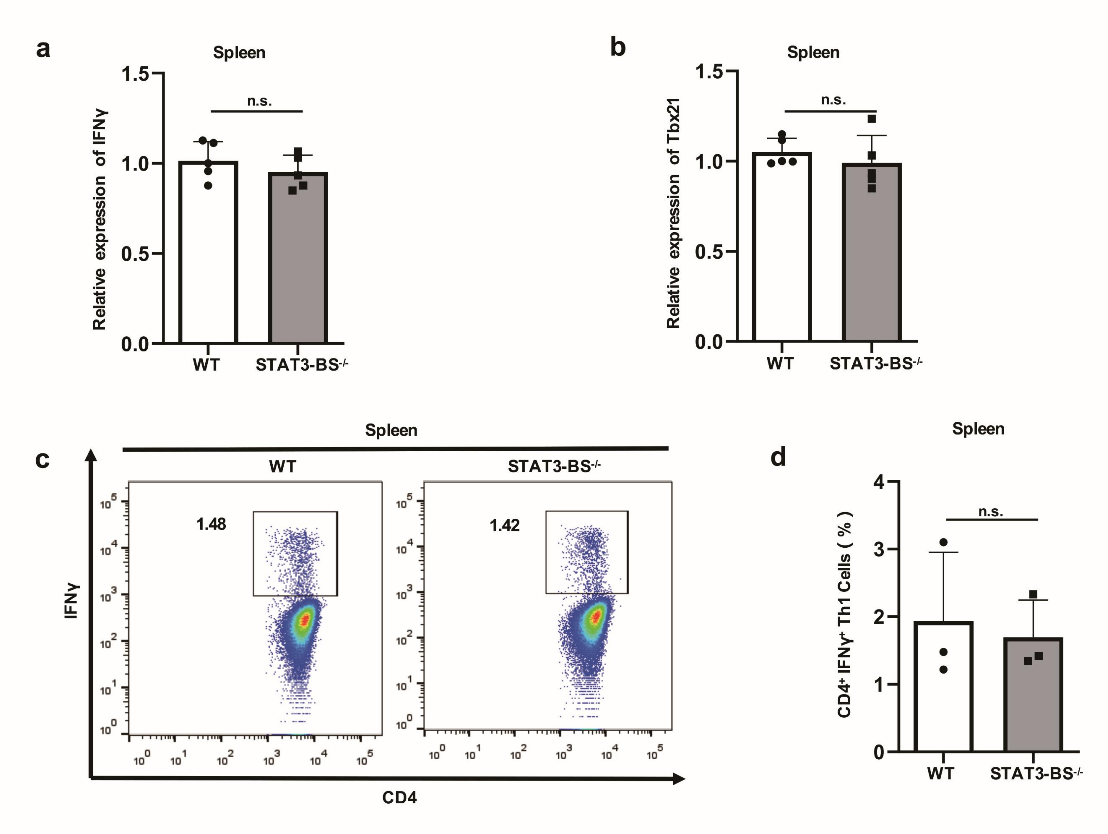

(a, b) Relative mRNA levels of *Tbx21* (a) and *IFNγ* (b) in the CD4$^+$ T cells of spleen from indicated mice were quantified by the RT–qPCR assay. (c, d) Flow cytometry analysis of the Th1 cell frequencies in splenic CD4$^+$ T cells. Means ± SEMs are shown, n = 5 biologically independent animals (a, b), n = 3 biologically independent animals (d).

Supplementary Fig. 3. STAT3-BS deficiency has no effect on Th2 cells in vivo.

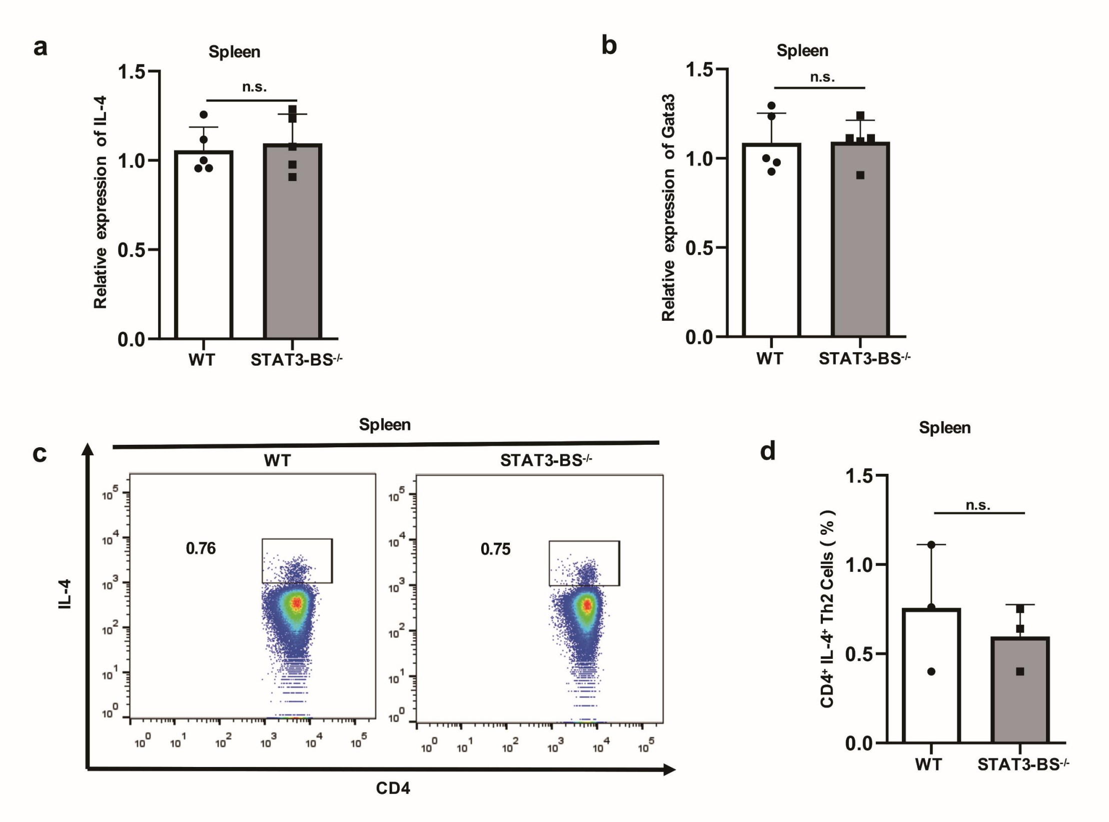

(a, b) Relative expression of *IL-4* (a) and *Gata3* (b) genes was quantified by RT–qPCR (a, b). (c, d) Flow cytometry analysis of the frequencies of Th2 cells in splenic $CD4^+$ T cells. Means ± SEMs are shown, n = 5 biologically independent animals (a, b), n = 3 biologically independent animals (d).

Supplementary Fig. 4 STAT3-BS deficiency has no impact on ILC3s in vivo.

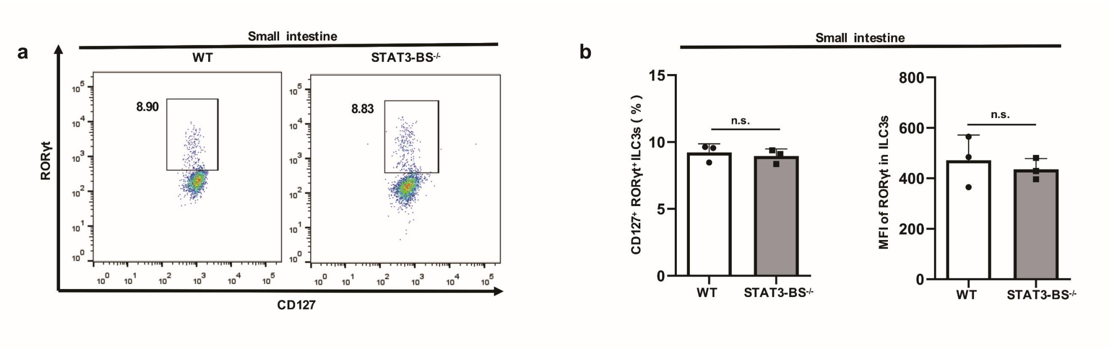

(a, b) Flow cytometry analysis of the CD127^{+} RORγt^{+} ILC3 frequencies in the CD127^{+} CD45^{+} Lin^{-} lymphocyte population and RORγt MFI in ILC3s from the small intestine of WT and STAT3-BS$^{-/-}$ mice. Means ± SEMs are shown, n = 3 biologically independent animals (b).

Supplementary Fig. 5. STAT3-BS deletion in RORCE2 has no effect on Th1 cell polarization in vitro.

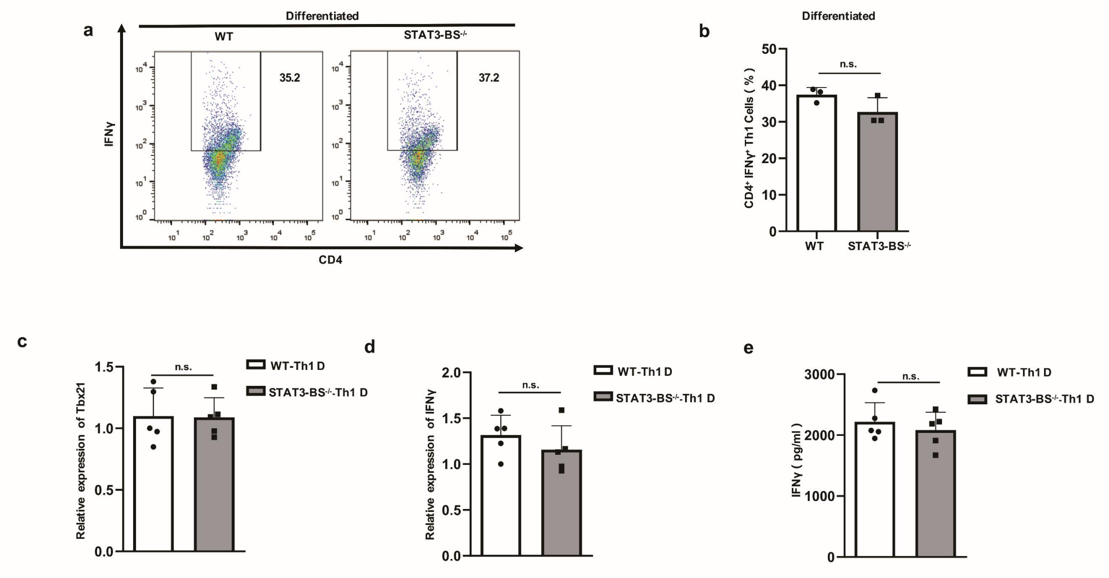

Splenic naïve $CD4^+$ T cells from the indicated mice were cultured in Th1-polarizing conditions for 3 days. (a, b) Flow cytometry analysis of the Th1 cell frequencies in the $CD4^+$ T cell population. (c, d) Relative mRNA levels of *Tbx21* (c) and *IFNγ* (d) in Th1-polarized cells. (e) An ELISA was used to measure IFNγ production in the culture supernatants. Means ± SEMs are shown, n = 3 biologically independent animals (b), n = 5 biologically independent animals (c-e). D = differentiation.

Supplementary Fig. 6. STAT3-BS deletion in RORCE2 has no effect on Th2 cell polarization in vitro.

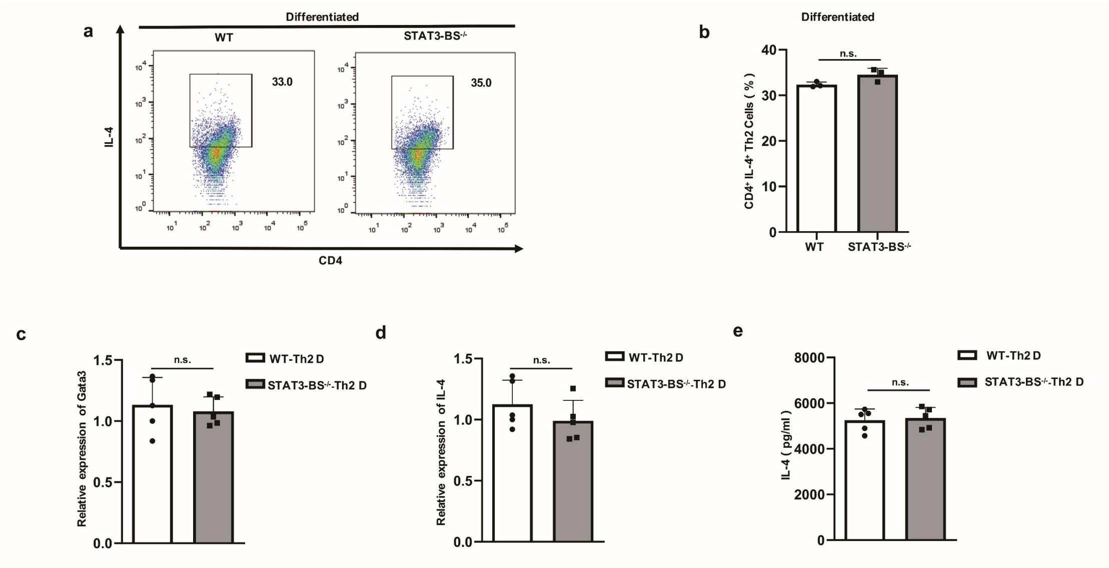

Splenic naïve $CD4^+$ T cells from the indicated mice were cultured in Th2-polarizing conditions for 3 days. (a, b) Flow cytometry analysis of the Th2 cell frequencies in the $CD4^+$ T cell population. (c, d) Relative mRNA levels of the *Gata3* (c) and *IL-4* (d) genes in Th2-polarized cells. (e) An ELISA was used to measure IL-4 production in the culture supernatants. Means ± SEMs are shown, n = 3 biologically independent animals (b), n = 5 biologically independent animals (c-e). D = differentiation.

Supplementary Fig. 7 The Th1 and Th2 cell frequencies in EAE-induced mice.

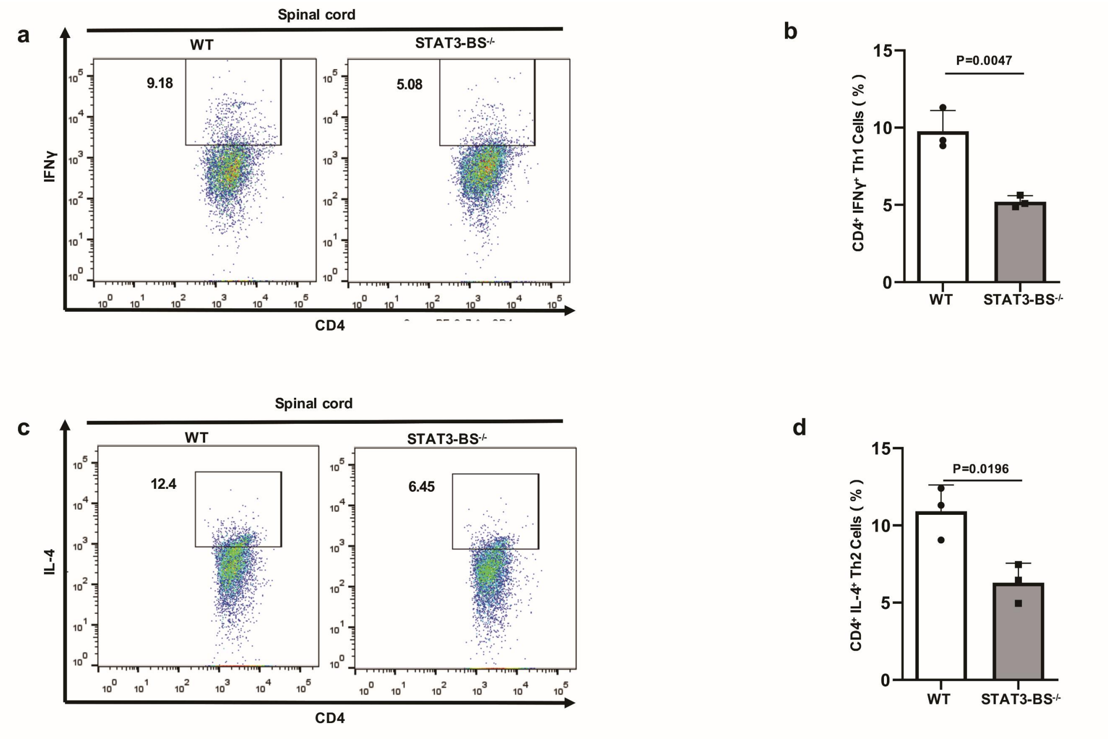

(a-d) Flow cytometry analysis of mononuclear cells from the spinal cord of EAE-induced mice at 30 days after immunization. The gated CD4^{+} T cells were further evaluated for Th1 (a, b) and Th2 (c, d) cell frequencies. Means ± SEMs are shown, n = 3 biologically independent animals (b, d).

Supplementary Fig. 8. Deletion of STAT3-BS in RORCE2 reduced the frequencies of TCRγδ$^+$ IL-17A$^+$ γδT17 cell.

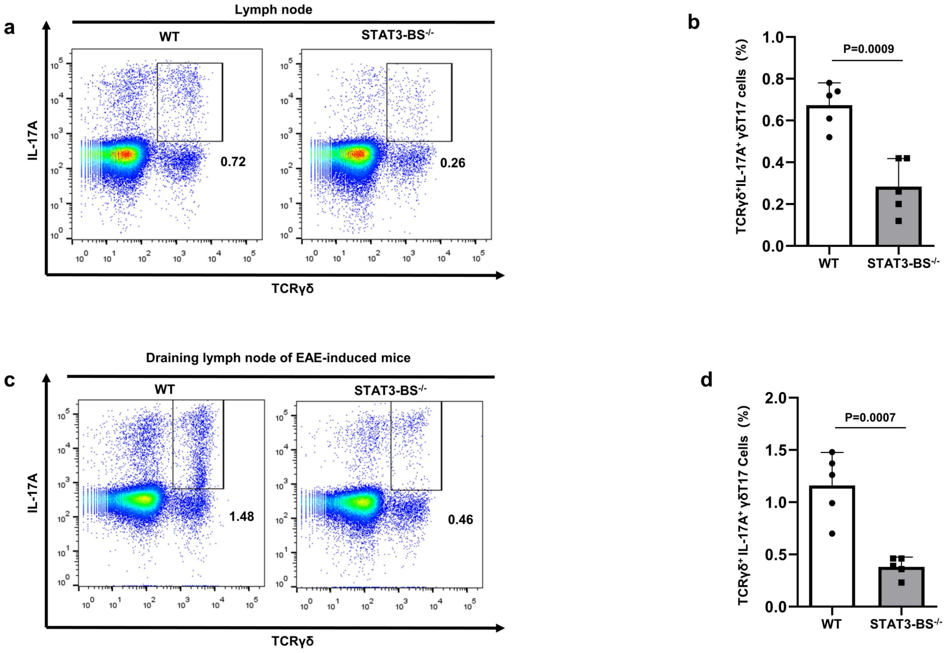

(a-b) Flow cytometric analysis of TCRγδ$^+$ IL-17A$^+$γδT17 cells in the CD3$^+$ lymphocyte population from the lymph nodes of STAT3-BS$^{-/-}$ and WT mice. (c-d) Flow cytometric analysis of TCRγδ$^+$ IL-17A$^+$ γδT17 cells in the CD3$^+$ lymphocyte population from the draining lymph nodes of STAT3-BS$^{-/-}$ and WT mice with induced EAE. Means ± SEMs are shown, n = 5 biologically independent animals (b, d).

Supplementary Fig. 9. SOX-5-BS deletion in RORCE2 reduced the chromatin accessibility of RORCE2.

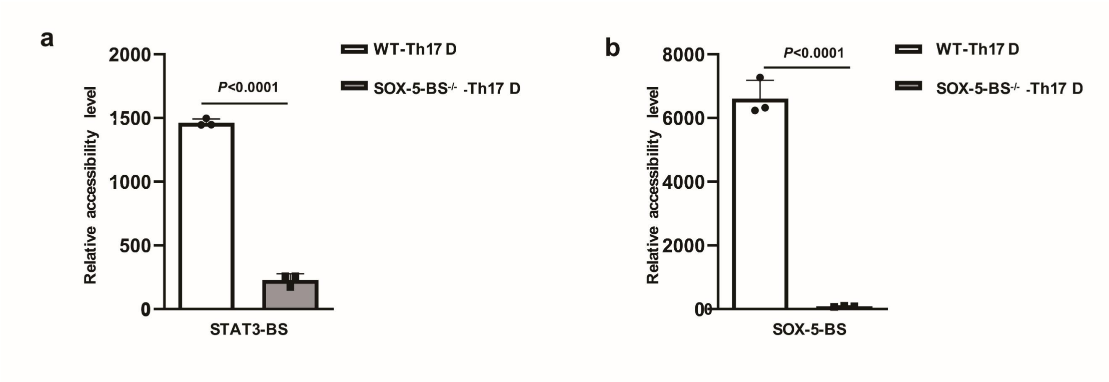

Chromatin accessibility assays were performed on SOX-5-BS (a) and STAT3-BS (b) in RORCE2 of the indicated cells. Means ± SEMs are shown, n = 3 biologically independent animals (a–b). D = differentiation.

Supplementary Fig. 10. Comparable BRG1 protein levels in Th1-, Th2-, Th17- and Treg-polarized cells.

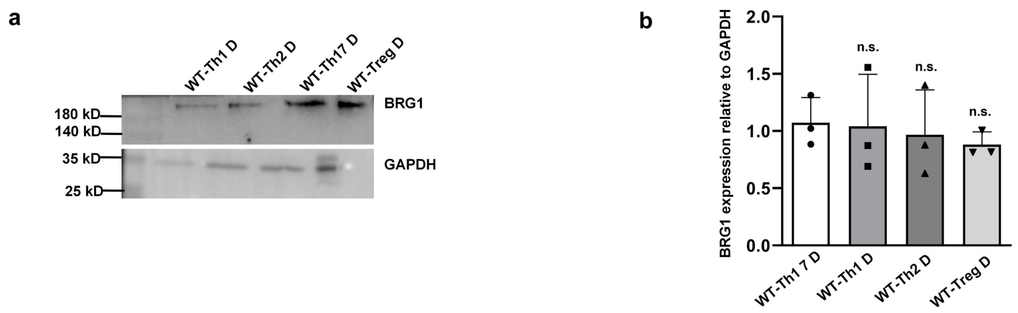

(a-b) The protein expression levels of BRG1 and GAPDH in Th1-, Th2-, Th17- and Treg-polarized cells were measured by Western blotting, and the unedited blot/gel images were shown in Supplementary Fig. 11d. Quantitative measurement of BRG1 band was performed by ImageJ software. Means ± SEMs are shown, n = 3 biologically independent animals (b). D = differentiation.

Supplementary Fig. 11. Uncropped and unedited blot/gel images.

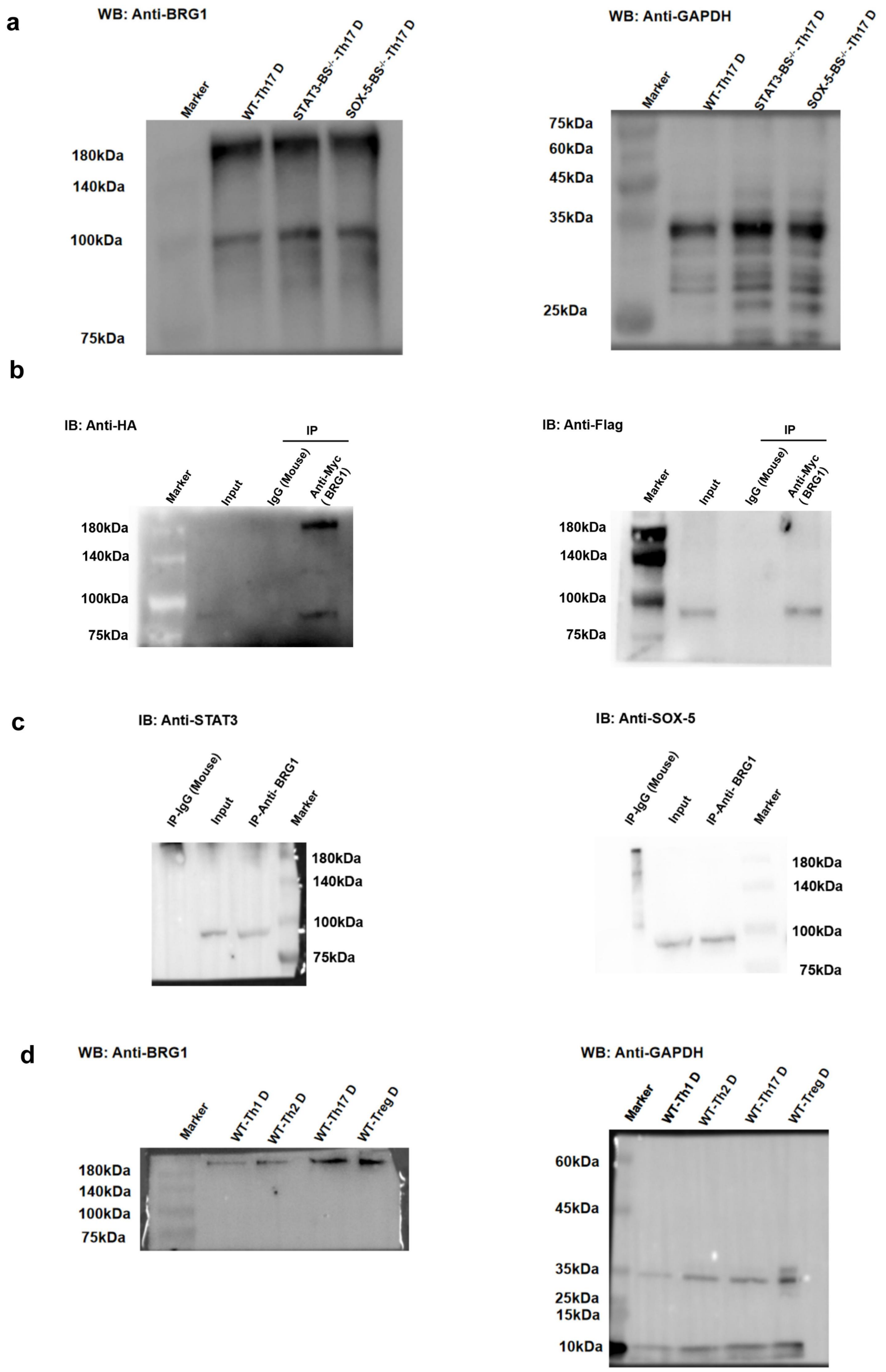

(a)Uncropped and unedited blot/gel images of Fig. 5c. (b) Uncropped and unedited blot/gel images of Fig. 5f. (c) Uncropped and unedited blot/gel images of Fig. 5g. (d) Uncropped and unedited blot/gel images of supplementary Fig. 10a.

Supplementary Fig. 12. Gating strategies used for frequency analysis.

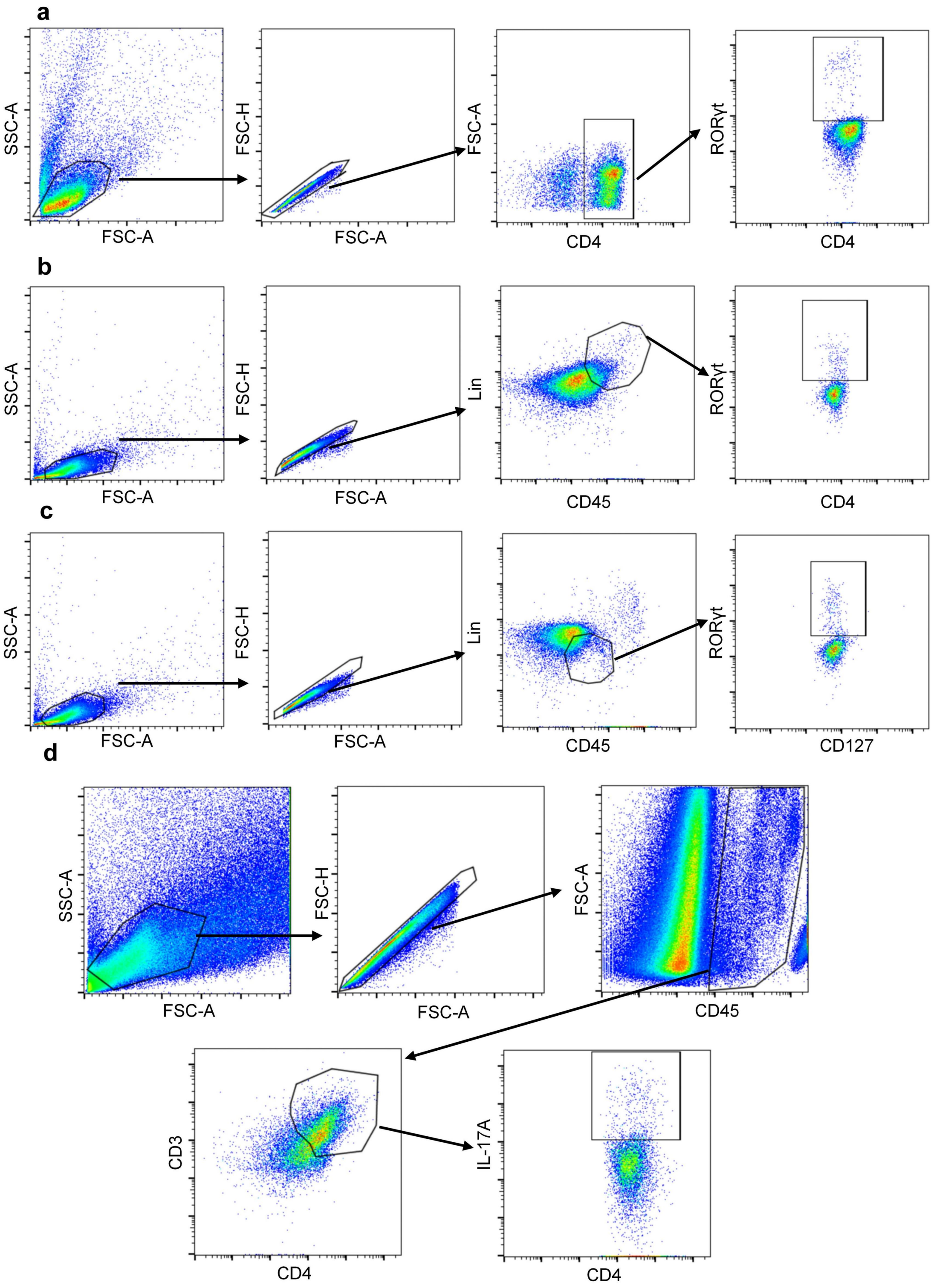

(a) Gating strategy to determine the percentage of CD4+ RORγt+ Th17 cells in splenic CD4+ T cells presented on Fig. 2b and the same strategy was used in Fig. 2e, Fig. 3a, supplementary Fig. 2c, supplementary Fig. 3c, supplementary Fig 5a, supplementary Fig 6a. (b) Gating strategy to

determine the percentage of RORγt^{+} Th17 in CD4^{+} CD45^{+} Lin^{+} lymphocyte population from LP of small intestine presented on Fig. 2g and the same strategy was used in Fig. 2i. (c) Gating strategy to determine the percentage of CD127^{+} RORγt^{+} ILC3 cells in the CD45^{+} Lin^{-} lymphocyte population of the lamina propria presented on Supplementary Fig. 4. (d) Gating strategy to analyze the percentage of CD4^{+} IL-17A^{+} Th17 cells in CD45^{+} CD3^{+} CD4^{+} T cells of the spinal cord of indicated mice presented on Fig. 4c and the same strategy was used in Supplementary Fig 7a and b.

Supplementary Table 1. Primers for ChIP–qPCR analysis.

Primer name	Primer name	Primer sequence (5´ to 3´)
STAT3-BS	STAT3-BS_F	5-GGGTAGGATGGACAGCTTCA-3
	STAT3-BS _R	5-AACAACTGTACACTCACCTTAG-3
SOX-5-BS	SOX-5-BS _F	5-TCCACTATGTTCCCACCAC-3
	SOX-5-BS _R	5-GTCAGCACGGAGGATTGTT-3
STAT3-SOX-5-BS	STAT3-SOX-5-BS_F	5-TCCACTATGTTCCCACCAC-3
	STAT3-SOX-5-BS _R	5-AACAACTGTACACTCACCTTAG-3

Supplementary Table 2. Primers for RT–qPCR

Gene name	Primer name	Primer sequence (5´ to 3´)
RORγt	RORγt _F	5-CGAGATGCTGTCAAGTTTGG-3
	RORγt _R	5-CACTTGTTCCTGTTGCTGCT-3
IL-17A	IL-17A _F	5-TCCAGAAGGCCCTCAGACTA-3
	IL-17A _R	5-TCAGGACCAGGATCTCTTGC-3
Tbx21	Tbx21_F	5-GCCAGGGAACCGGTTATATG-3
	Tbx21 _R	5-GACGATCATCTGGGTCACAT-3
Gata3	Gata3_F	5-AAGCTCAGTATCCGCTGACG-3
	Gata3_R	5-GTTTCCGTAGTAGGACGGGAC-3
IL-4	IL-4_F	5-CATCCTGCTTCACCAGAGAT-3
	IL-4_R	5-CATCGAAAAGCCCGAAAGAG-3
IFNγ	IFNγ_F	5-AGGCGAAAAAGGATGCATTC-3
	IFNγ-R	5-GACTCCTTTTCCGCTTCCT-3
GAPDH	GAPDH _F	5-ACAGCCGCATCTTCTTGTGCAGTG-3
	GAPDH _R	5-GGCCTTGACTGTGCCGTTGAATTT-3
