# Supplementary material for: STAT3 and SOX-5 induce BRG1-mediated chromatin remodeling of RORCE2 in Th17 cells
Source: Commun Biol. 2024 Jan 3;7:10. doi: 10.1038/s42003-023-05735-9 (PMC10764326; doi:10.1038/s42003-023-05735-9)
Supplement: Supplementary file 2 — Description of Additional Supplementary Files [file 42003_2023_5735_MOESM2_ESM.pdf]

Description of Additional Supplementary Files

File name: Supplementary Data 1
Description: The source data behind the figures in the paper.
