# Supplementary material for: STAT3 and SOX-5 induce BRG1-mediated chromatin remodeling of RORCE2 in Th17 cells
Source: Commun Biol. 2024 Jan 3;7:10. doi: 10.1038/s42003-023-05735-9 (PMC10764326; doi:10.1038/s42003-023-05735-9)
Supplement: Supplementary file 4 — Reporting Summary [file 42003_2023_5735_MOESM4_ESM.pdf]

Corresponding author(s): Yi Tian(COMMSBIO-23-2859A)

Last updated by author(s): Dec 13, 2023

Reporting Summary

Nature Portfolio wishes to improve the reproducibility of the work that we publish. This form provides structure for consistency and transparency in reporting. For further information on Nature Portfolio policies, see our Editorial Policies and the Editorial Policy Checklist.

Statistics

For all statistical analyses, confirm that the following items are present in the figure legend, table legend, main text, or Methods section.

n/a	Confirmed	
☐	☒	The exact sample size (*n*) for each experimental group/condition, given as a discrete number and unit of measurement
☐	☒	A statement on whether measurements were taken from distinct samples or whether the same sample was measured repeatedly
☐	☒	The statistical test(s) used AND whether they are one- or two-sided<br>*Only common tests should be described solely by name; describe more complex techniques in the Methods section.*
☒	☐	A description of all covariates tested
☒	☐	A description of any assumptions or corrections, such as tests of normality and adjustment for multiple comparisons
☐	☒	A full description of the statistical parameters including central tendency (e.g. means) or other basic estimates (e.g. regression coefficient) AND variation (e.g. standard deviation) or associated estimates of uncertainty (e.g. confidence intervals)
☐	☒	For null hypothesis testing, the test statistic (e.g. *F*, *t*, *r*) with confidence intervals, effect sizes, degrees of freedom and *P* value noted<br>*Give P values as exact values whenever suitable.*
☒	☐	For Bayesian analysis, information on the choice of priors and Markov chain Monte Carlo settings
☒	☐	For hierarchical and complex designs, identification of the appropriate level for tests and full reporting of outcomes
☒	☐	Estimates of effect sizes (e.g. Cohen's *d*, Pearson's *r*), indicating how they were calculated

Our web collection on statistics for biologists contains articles on many of the points above.

Software and code

Policy information about availability of computer code

Data collection	Flow cytometry measurments were performed on BD FACSCanto II instrument and BD FACSCanto cell analyzer. QPCR measurments were performed on BIO-RAD C1000 Touch and data was collected via CFX96TM Real-Time System. Tissue section staining measurments were performed on PreciPoint M8 Microscope. Western blotting was performed on BIO-RAD Gel Doe XR+. ELISA was performed on BIO-RAD iMark. Dual luciferase reporter assays were performed on Promega GloMax 20/20.
Data analysis	Microsoft office Excel 2021 ,Microsoft office word 2021,GraphPad Prism 8, FlowJo 10.0.7, BD FACSCanto cell analyzer were used for data analysis.

For manuscripts utilizing custom algorithms or software that are central to the research but not yet described in published literature, software must be made available to editors and reviewers. We strongly encourage code deposition in a community repository (e.g. GitHub). See the Nature Portfolio guidelines for submitting code & software for further information.

Data

Policy information about availability of data

All manuscripts must include a data availability statement. This statement should provide the following information, where applicable:

- Accession codes, unique identifiers, or web links for publicly available datasets
- A description of any restrictions on data availability
- For clinical datasets or third party data, please ensure that the statement adheres to our policy

All data generated and supporting the findings of our study are available within the paper and its Supplementary Information.

Research involving human participants, their data, or biological material

Policy information about studies with human participants or human data. See also policy information about sex, gender (identity/presentation), and sexual orientation and race, ethnicity and racism.

Reporting on sex and gender	*Use the terms sex (biological attribute) and gender (shaped by social and cultural circumstances) carefully in order to avoid confusing both terms. Indicate if findings apply to only one sex or gender; describe whether sex and gender were considered in study design; whether sex and/or gender was determined based on self-reporting or assigned and methods used. Provide in the source data disaggregated sex and gender data, where this information has been collected, and if consent has been obtained for sharing of individual-level data; provide overall numbers in this Reporting Summary. Please state if this information has not been collected. Report sex- and gender-based analyses where performed, justify reasons for lack of sex- and gender-based analysis.*
Reporting on race, ethnicity, or other socially relevant groupings	*Please specify the socially constructed or socially relevant categorization variable(s) used in your manuscript and explain why they were used. Please note that such variables should not be used as proxies for other socially constructed/relevant variables (for example, race or ethnicity should not be used as a proxy for socioeconomic status). Provide clear definitions of the relevant terms used, how they were provided (by the participants/respondents, the researchers, or third parties), and the method(s) used to classify people into the different categories (e.g. self-report, census or administrative data, social media data, etc.) Please provide details about how you controlled for confounding variables in your analyses.*
Population characteristics	*Describe the covariate-relevant population characteristics of the human research participants (e.g. age, genotypic information, past and current diagnosis and treatment categories). If you filled out the behavioural & social sciences study design questions and have nothing to add here, write "See above."*
Recruitment	*Describe how participants were recruited. Outline any potential self-selection bias or other biases that may be present and how these are likely to impact results.*
Ethics oversight	*Identify the organization(s) that approved the study protocol.*

Note that full information on the approval of the study protocol must also be provided in the manuscript.

Field-specific reporting

Please select the one below that is the best fit for your research. If you are not sure, read the appropriate sections before making your selection.

☒ Life sciences ☐ Behavioural & social sciences ☐ Ecological, evolutionary & environmental sciences

For a reference copy of the document with all sections, see nature.com/documents/nr-reporting-summary-flat.pdf

Life sciences study design

All studies must disclose on these points even when the disclosure is negative.

Sample size	Sample sizes were chosen on the basis of preliminary data from at least two pilot experiments and of previously published results in the literature.
Data exclusions	No data were excluded.
Replication	All experiments were repeated at least twice, with similar results.
Randomization	We did not use any randomization for experiments of STAT3-BS KO and WT mice. All mice of same background, similar age and sex were used in all experiments.
Blinding	The investigators were not blinded to group allocation during the experiments and outcome assessments because the data analyses were based on objectively measurable data.

Reporting for specific materials, systems and methods

We require information from authors about some types of materials, experimental systems and methods used in many studies. Here, indicate whether each material, system or method listed is relevant to your study. If you are not sure if a list item applies to your research, read the appropriate section before selecting a response.

Materials & experimental systems

n/a	Involved in the study
☐	☒ Antibodies
☐	☒ Eukaryotic cell lines
☒	☐ Palaeontology and archaeology
☐	☒ Animals and other organisms
☒	☐ Clinical data
☒	☐ Dual use research of concern
☒	☐ Plants

Methods

n/a	Involved in the study
☒	☐ ChIP-seq
☐	☒ Flow cytometry
☒	☐ MRI-based neuroimaging

Antibodies

Antibodies used	The following antibodies were used for the flow cytometry: CD3 (clone 145-2C11), CD4 (clones RM4-5, GK1.5), CD127 (clone A7R34), IFN-γ (clone XMG1.2), IL-17A (clone TC11-18H10.1), IL-4 (clone 1B11), RORγt (clone B2D), CD11b (clone M1/70), CD19 (clone 6D5), CD45 (clone 30-F11), Gr-1 (clone RB6-8C5) antibodies were purchased from eBioscience. The following antibodies were used for the ChIP-qPCR: SOX-5 (ab94396, Abcam), STAT3(124H6, Cell Signaling Technology), BRG1(AF300790, AiFang biological), H3K4me1 (ab176877, Abcam), H3K4me2 (ab32356, Abcam) and H3K27ac (ab4729, Abcam) antibodies. The following antibodies were used for the Co-IP and Immunoblotting (IB): Flag (M2, F3155, Sigma-Aldrich), Myc (9E10, ab32, Abcam), and HA (HA.C5, ab18181, Abcam).
Validation	Antibody validation was provided by manufacture's website (cell images) and/or data is provided by the paper.

Eukaryotic cell lines

Policy information about cell lines and Sex and Gender in Research

Cell line source(s)	293T and Hela cells were obtained from the American Type Culture Collection (ATCC).
Authentication	No further authentication was performed.
Mycoplasma contamination	The 293T and Hela cell lines were tested negative for mycoplasma contamination.
Commonly misidentified lines (See ICLAC register)	No commonly misidentified cell lines were used.

Animals and other research organisms

Policy information about studies involving animals; ARRIVE guidelines recommended for reporting animal research, and Sex and Gender in Research

Laboratory animals	All the experimental/control mice were maintained on a C57BL/6 background and were kept in standard cages (4–5 mice per cage) under specific pathogen–free conditions with food and water at stable room temperature and a 12/12-hour light/dark cycle. Mice aged 8 to 12 weeks were used for experiments without sex preference. We have complied with all relevant ethical regulations for animal use of the Institutional Animal Care and Use Committees of the Third Military Medical University.
Wild animals	No wild animals were used.
Reporting on sex	The mice used for experiments have no sex preference.
Field-collected samples	No field-collected samples were used.
Ethics oversight	All mouse experiments were performed in accordance with the guidelines of the Institutional Animal Care and Use Committees of the Third Military Medical University.

Note that full information on the approval of the study protocol must also be provided in the manuscript.

Flow Cytometry

Plots

Confirm that:

☒ The axis labels state the marker and fluorochrome used (e.g. CD4-FITC).

☒ The axis scales are clearly visible. Include numbers along axes only for bottom left plot of group (a 'group' is an analysis of identical markers).

☒ All plots are contour plots with outliers or pseudocolor plots.

☒ A numerical value for number of cells or percentage (with statistics) is provided.

Methodology

Sample preparation	Sample preparation is described in methods.
Instrument	BD FACSCanto II
Software	BD FACSCanto cell analyzer was used for data collection and FlowJo 10.0.7 was used for data analysis.
Cell population abundance	Data on the abundance of relevant cell population are provided in the paper.
Gating strategy	1. For analysis of Th17, Th1 or Th2 cells in the spinal cord mononuclear cells of EAE-induced WT and STAT3-BS KO mice, cells were gated on single cells in FCS/SSC gate, followed by exclusion of doublets in both FSC-A/FSC-H and SSC-A/SSC-H parameters, followed by gating on CD45+CD3+ .<br>2. For analysis of Th1, Th2 or Th17 cells in the spleen of WT and STAT3-BS KO mice, cells were gated on lymphocytes in FCS/SSC gate, followed by exclusion of doublets in both FSC-A/FSC-H and SSC-A/SSC-H parameters, followed by gating on CD4+.<br>3. For analysis of polarized Th1,Th2 or Th17 cells from the spleen of WT and STAT3-BS KO mice, cells were gated on lymphocytes in FCS/SSC gate, followed by exclusion of doublets in both FSC-A/FSC-H and SSC-A/SSC-H parameters.<br>4. For analysis of ILC3 in the lamina propria lymphocytes of WT and STAT3-BS KO mice, cells were gated on lymphocytes in FCS/SSC gate, followed by exclusion of doublets in both FSC-A/FSC-H and SSC-A/SSC-H parameters, followed by gating on CD127+CD45+Lin-.<br>5. For analysis of Th17 in the lamina propria lymphocytes of WT and STAT3-BS KO mice, cells were gated on lymphocytes in FCS/SSC gate, followed by exclusion of doublets in both FSC-A/FSC-H and SSC-A/SSC-H parameters, followed by gating on CD45+Lin+CD4+.

☒ Tick this box to confirm that a figure exemplifying the gating strategy is provided in the Supplementary Information.
